# Securing access to a comprehensive diagnostic panel for children with suspected acute lymphoblastic leukemia: Results from the Mexico in Alliance with St. Jude “Bridge Project”

**DOI:** 10.3389/fonc.2023.1286278

**Published:** 2024-01-15

**Authors:** Paola Friedrich, Nataly Mercado, Naomi Echeandia-Abud, Karla Guerrero-Gomez, Margarita González-Zamorano, Mayra Ivette López-Ruíz, Claudia Selene Portillo-Zavala, Laura Dolores García-Segura, Mariana Reynoso-Gutiérrez, Norma Araceli López-Facundo, Daniela Cárdenas-Pedraza, María Guadalupe Valois-Escamilla, Alma Beatriz Mera-González, Daniela Covarrubias-Zapata, Lilia Adriana Vollbrechtshausen-Castelán, José de Jesús Loeza-Oliva, Sergio Antonio Garay-Sánchez, Julio Moreno-Serrano, Patricia Mendoza-Sánchez, Paola Casillas-Toral, Antonio Sandoval-Cabrera, Itzel Gutiérrez-Martínez, Mariana Isabel Jiménez-Osorio, Daniela Arce-Cabrera, Dinora Virginia Aguilar-Escobar, Pablo Miguel González-Montalvo, Hugo Antonio Romo-Rubio

**Affiliations:** ^1^ Department of Global Pediatric Medicine, St. Jude Children’s Research Hospital, Memphis, TN, United States; ^2^ Casa de la Amistad para Niños con Cáncer, Institución de Asistencia Privada, I.A.P., Ciudad de México, Mexico; ^3^ Pediatric Oncology and Hematology Department, Hospital General con Especialidades “Juan María de Salvatierra”, La Paz, Mexico; ^4^ Pediatric Oncology Department, Hospital de Especialidades Pediátricas, Tuxtla Gutiérrez, Mexico; ^5^ Pediatric Oncology Department, Hospital Infantil de Especialidades de Chihuahua, Chihuahua, Mexico; ^6^ Pediatric Oncology Department, Hospital General de Celaya, Celaya, Mexico; ^7^ Hematology Department, Hospital General de León, León, Mexico; ^8^ Pediatric Oncology Department, Hospital Materno Infantil del Instituto de Seguridad Social del Estado de México y Municipios, Toluca, Mexico; ^9^ Pediatric Oncology and Hematology Department, Hospital para el Niño del Instituto Materno Infantil del Estado de México, Toluca de Lerdo, Mexico; ^10^ Pediatric Oncology Department, Hospital Infantil de Morelia "Eva Sámano de López Mateos", Morelia, Mexico; ^11^ Hematology Department, Hospital del Niño Desarrollo Integral de la Familia (DIF) Hidalgo, Pachuca de Soto, Mexico; ^12^ Pediatric Oncology Department, Centro Estatal de Oncología “Dr. Luis González Francis”, Campeche, Mexico; ^13^ Pediatric Oncology and Hematology Department, Hospital Infantil de Tamaulipas, Ciudad Victoria, Mexico; ^14^ Pediatric Oncology Department, Centro Estatal de Cancerología “Dr. Miguel Dorantes Mesa”, Xalapa, Mexico; ^15^ Diagnostic and Bood Bank Department, Hospital Infantil Teletón de Oncología, Querétaro, Mexico; ^16^ Pediatric Oncology and Hematology Department, Hospital Civil de Guadalajara “Dr. Juan I. Menchaca”, Guadalajara, Mexico; ^17^ Pediatric Oncology and Hematology Department, Hospital Pediátrico de Sinaloa, Culiacán, Mexico; ^18^ Pediatric Oncology Unit, Hospital General “Dr. Agustín O’Horán”, Mérida, Mexico

**Keywords:** acute lymphoblastic leukemia, pediatric, clinical characteristics, epidemiology, diagnostic panel, Mexico, multisite, consensus-derived

## Abstract

**Background:**

The “Bridge Project” is a Mexico in Alliance with St. Jude (MAS) initiative developed in 2019 to improve access, accuracy, and timeliness of specialized diagnostic studies for patients with suspected acute lymphoblastic leukemia (ALL). The project strategy relies on service centralization to improve service delivery, biological characterization, risk-group classification, and support proper treatment allocation.

**Methods:**

This is an ongoing prospective multisite intersectoral quality improvement (QI) project available to all patients 0-18 years of age presenting with suspected ALL to the 14 actively participating institutions in 12 Mexican states. Institutions send specimens to one centralized laboratory. From a clinical standpoint, the project secures access to a consensus-derived comprehensive diagnostic panel. From a service delivery standpoint, we assess equity, timeliness, effectiveness, and patient-centeredness. From an implementation science standpoint, we document feasibility, utility, and appropriateness of the diagnostic panel and centralized approach. This analysis spans from July 2019 to June 2023.

**Results:**

612 patients have accessed the project. The median age was 6 years (IQR 3-11), and 53% were males. 94% of the specimens arrived within 48 hours, which documents the feasibility of the centralized model, and 100% of the patients received precise and timely diagnostic results, which documents the effectiveness of the approach. Of 505 (82.5%) patients with confirmed ALL, 463/505 (91.6%) had B-cell ALL, and 42/505 (8.3%) had T-cell ALL. High-hyperdiploidy was detected by DNA index in 36.6% and hypodiploidy in 1.6%. 76.6% of the patients had conclusive karyotype results. FISH studies showed t(12;21) in 15%, iAMP21 in 8.5%, t(1;19) in 7.5%, t(4;11) in 4.2%, t(9;22) in 3.2%, del(9)(p21) in 1.8%, and TRA/D (14)(q11.2) rearrangement in 2.4%. Among B-cell ALL patients, 344/403 (85.1%) had Day 15 MRD<1% and 261/305 (85.6%) Day 84 MRD<0.01. For T-cell ALL patients 20/28 (71.4%) had Day 29 MRD<0.01% and 19/22 (86.4%) Day 84 MRD<0.01%.

**Conclusions:**

By securing access to a standardized consensus-derived diagnostic panel, the Bridge Project has allowed better characterization of childhood ALL in Mexico while producing unprecedented service improvements and documenting key implementation outcomes. We are using these results to inform iterative changes to the diagnostic panel and an associated treatment guideline (MAS-ALL18).

## 1 Introduction

Childhood cancer survival has significantly improved in high-income countries (HIC) over the last five decades (1, 2). For acute lymphoblastic leukemia (ALL), survival has improved for children 0-14 years old from 73% before the 1990s to 93% since 2010, and for adolescents 15 to 19 years old from 55% to 74% (3). These improvements result from the evolution of risk-adapted therapies, which now include clinical, biological, and genomic variables, and aim to maximize cure while minimizing toxicity (4). However, these advances have not been translated to low- and middle-income countries (LMIC), where most of the children with cancer live and where suboptimal health system performance, results in significant underdiagnosis and poor survival for thousands of children every year (5).

In 2004, Mexico pioneered financing innovations to respond to the increasing burden of childhood cancers among low-income families through the Fund for Protection Against Catastrophic Expenditures (“*Seguro Popular*”) (6). The program provided coverage for ALL starting in 2005, expanded to all childhood cancers by 2007, and accredited about 55 national hospitals to care for these children nationwide (7). However, by 2015, the documented 5-year net survival for childhood ALL remained below 60%, even after adjustment for a very high background childhood mortality (8). *Seguro Popular* prioritized increasing access to treatment through service decentralization. This approach, doubled the annual number of children with ALL accessing treatment (from 535 in 2005 to 1,070 in 2015), but the 5-year overall survival remained constant (at 61.8%; 95CI 60.8-62.9%), and wide gaps in survival and service delivery were documented (9, 10). The 5-year state-specific survival for children with ALL ranged from 43.7% to 74.7% throughout the country (9). Bottlenecks, inequities, and variations in quality across pediatric cancer centers prevailed, and rigidities in payment systems and treatment guideline accreditation delayed the adoption of innovations and hindered patient-centered, multi-site collaboration (6, 10). While *Seguro Popular* was specifically launched for the uninsured population, similarly low ALL survival was documented for the population with Social Security benefits and in multisector cohorts during the same period (11, 12). In 2020, *Seguro Popular* was suddenly dissolved, and the established national drug procurement systems were rapidly dismantled. Since then, the Mexican health system has been in constant redesign (6, 13). New models of care, health governance, and health financing are underway, but details on if, when, and how the health system will achieve new levels of system performance remain to be determined.

**Figure 1:**
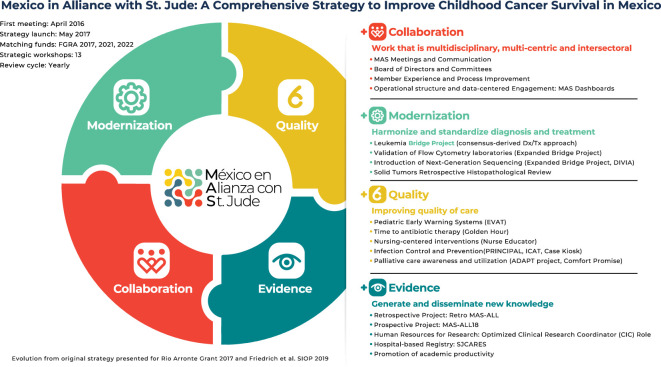
Mexico in Alliance with St. Jude (MAS) strategy.

Mexico in Alliance with St. Jude (MAS) emerged in 2016 as a multi-center, interdisciplinary, and intersectoral collaboration to improve the quality of care and survival for children and adolescents with cancer in Mexico through innovative education, treatment, and research strategies (14). It was formally launched as a cooperative group in 2017 by 11 founding healthcare institutions, with support from St. Jude Children’s Research Hospital’s “St. Jude Global” (St. Jude), “Casa de la Amistad para Niños con Cancer (CDLA),” and the Gonzalo Rio Arronte Foundation (FGRA) (15–17). Despite the aforementioned changes in the Mexican health system and the COVID-19 pandemic, MAS has grown to engage over 70 healthcare institutions in Mexico in collaboration and quality improvement activities and over 25 in modernization and evidence activities (see **Figure 1**). MAS has documented health system challenges to improve outcomes for children with ALL (18, 19), operationalized a model to increase access to specialized diagnostic testing for children with suspected ALL (20), developed and implemented an evidence-based consensus-derived ALL treatment guideline (21), expanded early detection of inpatient clinical deterioration utilizing pediatric early warning systems (22), improved time to antibiotic administration in patients with suspected febrile neutropenia (23, 24), helped sustain treatment continuity during the COVID-19 pandemic (25, 26), developed human resources for quality improvement and research (27), and nurtured collaboration with over 20 government agencies, professional organizations, and foundations (28).

This manuscript focuses on the “Bridge Project”, an ongoing, prospective, multisite, and intersectoral quality improvement (QI) project launched by MAS in 2019 to improve access, accuracy, and timeliness of specialized diagnostic studies for children with suspected ALL. It secures access to a consensus-derived diagnostic panel with the support and funding of local foundations and international collaborators. The diagnostic panel provides clinical teams with the essential information for precise ALL risk-group classification. Secondary aims include generating evidence for the feasibility and utility of carefully selected centralization practices, improving the understanding of the epidemiology of ALL in Mexico, and informing best practices for conducting multicenter and intersectoral collaborative work in diverse real-world settings. Although the project was not designed as a cancer outcomes study and cannot report on all clinically relevant characteristics, the results generated for ongoing service delivery and project oversight allow reporting on the clinical and epidemiological characteristics observed and the service and implementation outcomes obtained.

## 2 Methods

### 2.1 Project Design

The Bridge Project is an ongoing, prospective, multisite, and intersectoral quality improvement (QI) project that draws from improvement science to increase service equity, timeliness, effectiveness, and patient-centeredness and applies strategies of consensus development, prioritization, centralization, standardization, optimization, and training to meet these goals. It also draws from implementation science to document feasibility, utility, and appropriateness. It has continuously collected deidentified operational data with an improvement mindset since its activation in 2019.

### 2.2 Population

All consecutive patients 0-18 years of age presenting with suspected ALL to the participating MAS member institutions after their site-specific project launch (see “Project Context” and “Implementation Strategy” for details) were eligible to participate. Suspicion of ALL is determined locally by the pediatric hematology and/or oncology physician, utilizing clinical judgement and blood marrow morphology on light microscopy; a local immunophenotype or complementary diagnostic test is not required. This manuscript includes data gathered from all specimens collected between July 1^st^, 2019, and June 30^th^, 2023.

### 2.3 Project Development

From 2016 through 2019, MAS member institutions conducted weekly virtual meetings and eight strategic in-person meetings every six months. During these meetings, existing literature and local evidence were reviewed, multiple situational analyses were performed, and consensus on the preferred diagnostic panel, implementation strategy, and therapeutic approach were achieved (18, 19). Existing capacity and capability, logistics, preferences, and cost considerations were key for prioritization of the selected diagnostic panel, the sequence of training and external validation activities, and the focus on service centralization as a core improvement and implementation strategy. This experience led to the development, implementation, and early evaluation of MAS-ALL18, an evidence-based consensus-derived ALL treatment guideline, which most closely resembles Total XV, but incorporates several strategic evidence-based reductions to treatment intensity (21, 29).

### 2.4 Project Oversight

Participating institutions sign collaborating, billing, and data-sharing agreements with the three coordinating institutions: St. Jude Children’s Research Hospital (“St. Jude Global”; technical advisor), Hospital Infantil Teletón de Oncología (“HITO”; centralized laboratory), and Casa de la Amistad para Niños con Cancer (“CDLA”; administrative hub) (15, 16, 30). Participating institutions also obtain context-specific authorizations for participation and parents sign consent to collect and ship specimens following clinical institutional policies and procedures. Oversight of the project is jointly conducted by an advising committee comprised of five technical experts (PF, HR, PG, DA1, DA2), four members of the MAS steering committee (PF, HR, PG, LA), and two administrative staff (NE, NM). Project implementation meetings occur weekly and administrative oversight meetings occur monthly. The project obtained competitive grant funding from Gonzalo Río Arronte Foundation in 2017 and 2022 (17). Progress report for this external audience occurs at pre-set timelines every 8-12 months.

### 2.5 Project Context

**Figure 2:**
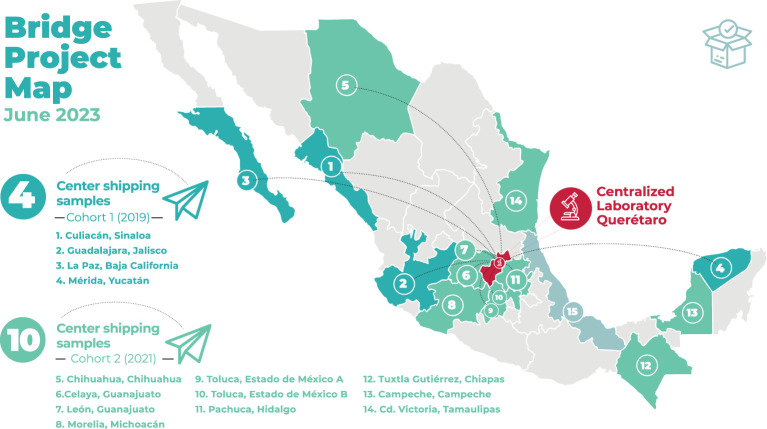
Geographic distribution of participating centers.

The project is actively running in fourteen MAS member institutions in twelve Mexican states (see **Table 1** and **Figure 2**). Participating institutions include general, pediatric, and specialty hospitals with pre-established/mature pediatric hematology and/or oncology (PHO) wards. Regarding eligibility and readiness, HITO was identified early on as the most suitable location for the centralized laboratory based on its existing equipment, human resources, procurement capability, leadership buy-in, and openness to complete thorough external validation and training activities (31, 32). Additional institutions were invited to participate if they were already actively participating in other MAS projects, verbalized interest and commitment to the project goals, formed a multidisciplinary team and engaged their leadership to sign the required collaborative agreements. Sites unable to sign or renew the collaborating agreements could participate in learning and knowledge-sharing activities (including in-person and virtual meetings) but could not ship specimens to the centralized laboratory.

**Table 1:** Population: bridge project participating centers, volume, and engagement.

Institution	Hospital Name	Cohort	Date of first shipment (n=16)	ALL Suspected (n=612)	ALL Confirmed (n=505)	MAS-ALL-18 start date (n=12)	Patients on MAS-ALL18 (n=349)
Lab	Hospital Infantil Teletón de Oncología (HITO) Querétaro, Querétaro	Centralized Laboratory					
1	Hospital Pediátrico de Sinaloa Culiacán, Sinaloa	1	July 2019	95	74	July 2019	58
2	Hospital General “Dr. Agustín O’Horán” Mérida, Yucatán	1	July 2019	107	89	July 2019	85
3*	Hospital General de Tijuana Tijuana, Baja California	NA*	August 2019	1	1	NA*	NA*
4*	Centro Estatal de Cancerología “Dr. Miguel Dorantes Mesa” Xalapa, Veracruz	NA*	August 2019 – February 2021	15	15	NA*	NA*
5	Hospital General con Especialidades “Juan María de Salvatierra” La Paz, Baja California Sur	1	February 2020	16	16	December 2021	5
6	Hospital Civil de Guadalajara “Dr. Juan I. Menchaca” Guadalajara, Jalisco	1	May 2020	130	112	June 2020	104
7	Hospital de Especialidades Pediátricas Tuxtla Gutiérrez, Chiapas	2	June 2022	41	26	January 2023	14
8	Hospital Infantil de Especialidades del Estado de Chihuahua Chihuahua, Chihuahua	2	January 2022	11	9	January 2022	9
9	Hospital General de Celaya Celaya, Guanajuato	2	February 2022	18	10	February 2022	10
10	Hospital General de León León, Guanajuato	2	February 2022	29	21	July 2022	14
11	Hospital para el Niño del Instituto Materno Infantil del Estado de México, Toluca, Estado de México	2	March 2022	33	31	NA*	NA*
12	Hospital Materno Infantil del Instituto de Seguridad Social del Estado de México y Municipios, Toluca, Estado de México	2	March 2022	10	9	March 2023	4
13	Hospital Infantil de Morelia “Eva Sámano de López Mateos” Morelia, Michoacán	2	March 2022	65	55	May 2022	40
14	Hospital del Niño DIF-Hidalgo Hidalgo, Pachuca	2	April 2022	32	30	NA*	NA*
15	Centro Estatal de Oncología “Dr. Luis González Francis” Campeche, Campeche	2	July 2022	6	4	October 2022	3
16	Hospital Infantil de Tamaulipas Ciudad Victoria, Tamaulipas	2	March 2023	3	3	May 2023	3

*NA, Not applicable; not actively participating in the Bridge Project.

### 2.6 Consensus-derived diagnostic panel

The consensus-derived diagnostic panel has been previously published and includes bone marrow morphology, immunophenotype, DNA index, karyotype (with analysis of 20 metaphases), fluorescence *in situ* hybridization, and minimal residual disease (MRD) evaluation by flow cytometry at two-time points (day 15, and/or day 29, and day 84) depending on the ALL lineage and specifications on the MAS-ALL18 or institutional treatment guideline (33). The flow cytometry diagnostic and MRD panels were developed in collaboration with and through external validation with Boston Children’s Hospital and St. Jude Children’s Research Hospital (31, 32). The diagnostic flow cytometry panel includes B lineage markers (CD20+, CD10+, CD19+), T lineage markers (CD3+, CD4+, CD8+, CD7+, CD5+), myeloid lineage markers (HLDR, CD15+, CD13+, CD117+, CD33+, CD16+), cytoplasmic and nuclear markers (TdT, myeloperoxidase, CD3+, CD79a, IgM) and non-specific lineage markers (CD81+, CD58+, CD34+, CD38+, CD99+). The B-cell ALL MRD panel includes B lineage markers and non-specific lineage markers (CD81+, CD20+, CD10+, CD19+, CD58+, CD34+, CD38+). The T-cell ALL MRD panel includes T lineage markers (CD3+, CD4+, CD7+, CD8+, CD5+), CD34+, and CD99+. The FISH panel for B-cell ALL includes KMT2A, ETV6/RUNX1, BCR/ABL, E2A/PBX1, and iAMP21 and the FISH panel for T-cell includes KMT2A, BCR/ABL, E2A/PBX1, TLX1, TLX3, CDKN2A, and TRA/D. See **Tables 2**, **3** for diagnostic panel results. Costs of the diagnostic panel are available in the Supplemental Material (see **Supplementary Resource 1**).

**Table 2:** Clinical and molecular characteristics observed.

Characteristic	Value, n (%)
Sex (n, %)	
Suspected ALL, Male (n=612)	325 (53.1%)
Confirmed ALL, Male (n=505)	268 (53.1%)
Age (years)	
Suspected ALL, Median (IQR) (n=612)	6 (3-11)
Confirmed ALL, Median (IQR) (n=505)	6 (3-11)
Final diagnosis (n=612)	
Acute lymphoblastic leukemia (ALL)	505 (82.5%)
Acute myeloid leukemia (AML)	42 (6.9%)
Other leukemias	4 (0.7%)
No evidence of leukemia	61 (10%)
Confirmed ALL, Immunophenotype (n=505)	
B cell ALL	463 (91.6%)
T cell ALL	42 (8.3%)
Confirmed ALL, Age category (years; n=505)	
<1	7 (1.4%)
1-10	347 (68.7%)
>=10	151 (29.9%)
Confirmed ALL, DNA Index (n=505)	
Abnormal (High-Hyperdiploid; >1.16)	185 (36.6%)
Abnormal (Hyperdiploid; 1.05 -1.16)	60 (11.9%)
Normal (Diploid; 0.9 - 1.05)	248 (49.1%)
Abnormal (Hypodiploid; <0.9)	8 (1.6%)
Not processed	4 (0.8%)
Confirmed ALL, Karyotype (n=505)	
Normal	131 (25.9%)
Abnormal	256 (50.7%)
No development	111 (22.0%)
Not processed	7 (1.4%)
Confirmed ALL, Abnormal Karyotype (n=256)*	
Hyperdiploid	86 (17.0%)
Complex karyotype	50 (9.9%)
t(1;19)	19 (3.8%)
del(12)(p12-13)	10 (2.0%)
iAMP dup(21)(q21q22)	9 (1.8%)
t (9;22)	10 (2.0%)
t(4;11)	5 (1.0%)
del(11)(q21q23)	4 (0.8%)
Type T/del(9)(p21)	3 (0.6%)
Other	60 (11.8%)
Confirmed ALL, FISH (n=505)	
Positive	364 (72.1%)
Negative	134 (26.5%)
Not processed	7 (1.4%)
Confirmed ALL, Positive FISH Results (n=364)*	
Gene gains	149 (29.5%)
t(12;21); ETV6-RUNX1	76 (15.0%)
iAMP21	43 (8.5%)
t(1;19); E2A/PBX1	38 (7.5%)
t(4;11); KMT2A	21 (4.2%)
t(9;22); BCR/ABL	16 (3.2%)
TRA/D(14) (q11.2)**	12 (2.4%)
CDKN2A del(9)(p21)**	9 (1.8%)

*The denominator for the percentages listed is total number of confirmed ALL (n=505).

**These are specific for T lineage ALL. Among the 42 patients with T-lineage, percentage increases to 28.67% and 21.4%, for TRA/D(14) (q11.2) and CDKN2A del(9)(p21), respectively.

**Table 3:** MRD results.

Total	MRD D15 n (%)	MRD D29 n (%)	MRD D84 n (%)
	n=407	n=37	n=327
*B cell ALL (n= 463 patients)	**n=403 (87%)**	**n=9 (1.9%)**	**n=305 (65.9%)**
*<0.01%*	197 (48.9%)	7 (77.8%)	261 (85.6%)
*≥0.01% - <1%*	147 (36.5%)	1 (11.1%)	21 (6.9%)
*≥1%*	48 (11.9%)	0 (0%)	7 (2.3%)
*No result available^*	11 (2.7%)	1 (11.1%)	16 (5.2%)
**T cell ALL (n= 42 patients)	**n= 4 (9.5%)**	**n= 28 (66.6%)**	**n= 22 (52.4%)**
*<0.01%*	2 (50%)	20 (71.4%)	19 (86.4%)
*>=0.01%*	2 (50%)	7 (25%)	2 (9.1%)
*No results available^*		1 (3.6%)	1 (4.5%)

*412 (88.9%) of 463 patients with B cell ALL had MRD performed at Day 15 or 29 and 305 (65.8%) at Day 84.

**32 (76.1%) of 42 patients with T cell ALL had MRD performed at Day 29 or 15 and 22 (52.4%) at day 84.

^Existing billing bases do not have MRD results available

Implementation strategy: MAS member institutions meeting eligibility/readiness criteria (see “Project context”) are invited to participate. The institutions form improvement teams and undergo QI training and coaching to standardize the diagnostic specimen collection, packing, handling, and shipment process. The teams develop a situational analysis, a block diagram, and a set of checklists to guide every step of the local process, follow a common theory of change, apply the measurement strategy, develop change ideas, and conduct and document Plan-Do-Study-Act (PDSA) cycles. Teams also learn to use standardized project forms (see “Data and Data Collection”). Finally, two members of each team complete a certificate-based training on the proper handling and shipping of biological specimens (“Online Biological Substance Category B IATA” by the Dangerous Goods International Training Center).

Before sending the first patient specimen, each team must develop and prototype a diagnostic specimen collection and shipment process that allows most specimens to arrive Monday through Friday at the centralized laboratory. To achieve this, each team must send at least five empty boxes on five different days (covering Monday through Friday). Teams may utilize existing or try out new couriers and can send additional empty boxes if a pattern and/or courier preference can’t be established. During this phase, teams typically make changes to their systems and processes, restructuring procedure days, times, and personnel to optimize their strategy. Specialty couriers allow Friday collection and Saturday arrival, but teams are asked to limit their use to serve unstable new patients due to cost considerations (specialty shipping costs are 4x baseline).

**Figure 3:**
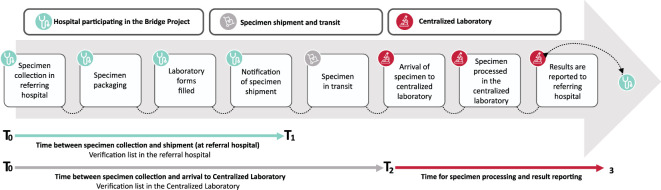
Process map and relevant times.

Once the institution is deemed ready, the team can start sending patient specimens for all consecutive patients that meet the project inclusion criteria (see “Population”). Teams collect the specimen, fill out the project forms, document relevant times (See **Figure 3**), ship the specimen, and alert the laboratory that a specimen is in transit. Upon receipt, HITO documents specimens’ arrival time and conditions utilizing a standardized checklist (see **Supplementary Resource 2**). The laboratory inspects and evaluates the condition of each tube/specimen upon arrival and labels each one as “adequate”, “conditioned”, or “rejected”. The threshold to label specimens as “conditioned” is low and most often includes non-critical clerical errors, hemolysis, or clotting, among others. However, all “adequate” and “conditioned” specimens have been processed and a “conditional” status has not been associated with challenges in interpretation. We have continued the three-tier categorization despite “conditional” labeling having little to no clinical consequence, as a mechanism to raise awareness and train participating institutions on the value of optimizing the pre-analytical sample conditions. If a specimen is rejected, typically due to critical delays, temperature, clerical errors, spills, or clotting clearly affecting sample quality, HITO contacts the treating physician immediately to request another specimen. Finally, HITO provides clinical results to the treating physician listed in the requisition within specific timelines (immunophenotype is reported within 48 hours from receipt date/time, FISH within six days, and karyotype within 26 days). The laboratory team at HITO is available to answer questions from the treating physician about the specimen, panel, studies, and clinical impressions. In addition, HITO provides feedback to the implementing team about arrival time, package, and specimen conditions shortly after every shipment. This allows the implementing teams to make real-time iterative improvements to their diagnostic specimen collection, handling, and shipment process. The multidisciplinary teams (pediatric oncologists, research coordinators, nurses, social workers, and laboratory technicians) from the participating hospitals hold weekly meetings to learn and guide QI activities.

The cost of the consensus-derived diagnostic panel has been covered by the 2017 and 2022 FGRA grants, which provide funding for over 1,000 patients with confirmed ALL. Detailed cost of the diagnostic panel is available in **Supplementary Resource 1**. CDLA covers the costs of diagnostic tests not included in the diagnostic panel, which are needed when identifying non-ALL diagnoses. St. Jude provides funding for project management, two laboratory technicians at the central laboratory, one research coordinator at each participant institution, training/certification for the shipment of biological specimens, supplies included in the shipping kits, and courier expenses. HITO and CLDA handle billing following national and institutional standards.

### 2.7 Data and Data Collection

All diagnostic specimens sent utilizing the described Bridge Project mechanisms are included in the operational database. Data is collected through standardized project forms (laboratory requisition, clinical summary, sharing data agreement, and shipment notification form). Clinical data to deliver patient care is collected and shared on a need-to-know basis following institutional and national policies and procedures. Only the treating physicians and the laboratory staff that receive, process, and interpret specimens have access to patient identifiers. Technical experts, steering committee, and project staff work exclusively with deidentified data.

### 2.8 Data analysis

Operational definitions for service and implementation outcomes are provided in **Table 4** and **Figure 3**. Descriptive statistics were utilized to summarize patient characteristics.

**Table 4:** Service and Implementation outcomes.

Service outcome of interest	Definition	Result	Associated Implementation outcome	Interpretation
**Timeliness**	**% of specimens arriving in ≤48hrs;** based on time between specimen collection and arrival to centralized laboratory; from T_0_ to T_2_ in **Figure 3**	**94%** (n=612)	**Feasibility**	**Centralization of specialized diagnostic tests is feasible in Mexico;** logistics pose a challenge but knowledge generation at institutions and knowledge transfer between institutions is feasible to meet the target.
**Effectiveness**	**% of patients with precise and timely diagnostic confirmation**; based on the number of complete* diagnostic reports provided to treating physicians over the total number of patient specimens sent	**100%** (n=612)	**Utility**	**The workflows established meet the needs of patients with and without ALL.** **The results are useful to treating physicians** making decisions for patients with and without ALL.
**Patient-centeredness**	**% of ALL patients for whom all necessary elements for precise risk stratification at diagnosis were reported**; based on the day 0 elements in the consensus-derived diagnostic panel and institutional protocol	**98.2%** (n=505)	**Appropriateness**	**The consensus-derived diagnostic panel** meets the needs of the patients with confirmed ALL; it **allows precise and comprehensive risk stratification.**
**Equity**	% of consecutive patients with suspected ALL who accessed the panel**; based on the number of patients with suspected ALL for whom a specimen was sent over the total number of patients with suspected ALL during the same period	**93.9%** (n=652)	**Equity**	**Secured financing and centralization** of specialized diagnostic tests **allows equitable access and offers same chance of risk-adapted therapy** to all children with suspected ALL, regardless of geographic location, health sector, or ability to pay

*The definition of a complete diagnostic report varies depending on the morphology and immunophenotype identified. For a patient with ALL, it is the consensus-derived day 0 diagnostic panel. For non-ALL, additional studies were only sent if deemed appropriate for the suspected diagnosis. Not all patients accessed MRD evaluation (see text for details).

**We have identified about 40 patients who presented to MAS member institutions but did not enter the Bridge Project. None were excluded due to their geographic location or ability to pay. Patients were excluded due severe clinical deterioration, weekend presentation with urgent need to start therapy, lack of sufficient sample, or immediate transfer to a hospital with access to specialized diagnostic studies.

### 2.9 Ethics

This is a quality improvement project and was exempt from IRB review.

## 3 Results

### 3.1 Population (Centers)

As seen in **Table 1**, sixteen institutions have joined the Bridge Project since it launched and fourteen (88%) are actively participating. Thirteen of the fourteen active institutions are public hospitals serving the needs of predominantly low to lower-middle income patients without social security or employment-based benefits. Four institutions have been actively shipping specimens to the centralized laboratory since 2019 and 2020 (the pilot, “Cohort 1”), ten joined the expansion cohort (“Cohort 2) in August 2021, and two (12.5%) were unwilling or unable to continue shipping specimens (“NA”). One institution is close to the United States border (in Tijuana) and was able to procure access to a diagnostic panel in the United States with local foundation support and one institution (in Xalapa) has been unable to sign the new version of the required agreements due to changes in the administrative officials. The geographic distribution of the fourteen actively participating institutions and the centralized laboratory are shown in **Figure 2**. A total of 612 cases have been sent for review for suspected ALL to the centralized laboratory, and ALL has been confirmed in 505 (82.5%). Twelve active institutions (86%) are standardizing and optimizing their treatment approach by adopting MAS-ALL18, an evidence-based consensus-derived treatment guideline. Four institutions adopted the diagnostic panel and treatment guideline at the same time; the others did it sequentially. As seen in **Table 1**, eleven institutions see <35 new ALL patients per year.

### 3.2 Population (Patients)

**Table 2** displays the clinical and epidemiological characteristics available through the Bridge Project. Among 612 patients with suspected ALL, the median age was 6 years, and 53.1% were males. Ten percent of patients did not have leukemia, 6.9% had acute myeloid leukemia, and 0.7% had other leukemias. These findings are within the expected range when sending specimens of suspected ALL based on clinical presentation and morphology without additional confirmatory tests. Most patients with confirmed ALL had B-cell (91.6%) and were in the 1-10 age group.

### 3.3 Molecular characteristics

DNA index, karyotype, and FISH results were available for 501 (99.2%), 498 (98.6%), and 498 (98.6%) of patients, respectively. Hypodiploidy was identified in 1.6% of patients by DNA index. Relevant mutations were more frequently identified through FISH (72.1%) than with karyotype (50.7%). The frequency of t(12;21) by FISH was 15%, t(1;19) was 7.5%, t(4;11) was 4.2%, and t(9;22) was 3.2%. The frequency of iAMP21 was 1.8% by karyotype and 8.5% by FISH. Among the 39 patients with T-cell ALL, the frequency increases to 30.7% and 23.1%, for TRA/D(14) (q11.2) and CDKN2A del(9)(p21), respectively.

### 3.4 MRD results

MRD results were available for 403 (87%) of 463 patients with B cell ALL at Day 15 and 305 (65.9%) on Day 84, and for 28 (66.6%) of 42 patients with T cell ALL on Day 29 and 22 (52.4%) at day 84. MAS-ALL18 and Total XV utilize MRD Lite at Day 15 to evaluate early response to therapy in patients with B-cell ALL. As shown in **Table 3**, 85.1% of patients B-cell ALL had MRD <1% at Day 15. Furthermore, 48.9% of patients with B-cell ALL had MRD<0.01% at day 15, 77.8% at day 29, and 85.6% at day 84. The numbers on day 29 are small because, in MAS-ALL18, MRD is not routinely sent on day 29 for patients with B-cell ALL; it is only sent if the treating physician is concerned about induction failure to support distinguishing blasts and hematogones. Day 29 results for patients with B-cell ALL reflect the use of Total XV as the institutional protocol or concerns for induction failure. For patients with T-cell ALL, 71.4% had MRD<0.01% at day 29 and 86.4% at day 84. The observed frequency of MRD evaluations was lower than anticipated. A sub-analysis of the causes for missed MRD showed that the main challenge to completing MRD evaluation was death prior to the established MRD evaluation timepoint, which occurred in 3% of B-cell ALL patients before day 15 and 8.2% of patients before day 84, and 33% of T-cell ALL patients before day 29 and 21.4% of patients before day 84. The second most common challenge was the institutional guideline not utilizing the MRD timepoint, which was specific to day 84 and occurred in 9.1% of patients with B-cell ALL and 7.1% of patients with T-cell ALL. Additional reasons to miss MRD evaluation included transfers to another facility, clinical instability, lack of coordination to send sample, and treatment abandonment. Details are provided in **Supplementary Resource 3**.

### 3.5 Service improvement and implementation results

**Table 4** summarizes the service outcomes of interest as well as their associated implementation outcome and interpretation. Most (94%) of specimens have arrived within 48 hours of collection and 100% of patients received a diagnosis, regardless of whether they had ALL or not. Less than 2% of specimens had to be rejected and real-time communication allowed for all patients with rejected specimens the opportunity for timely shipment of a second specimen. Finally, we achieved 93.9% equitable access. The equity measure is defined by the percentage of consecutive patients with suspected ALL who access the panel. We have identified that up to 40 patients who presented to MAS member institutions but did not enter the Bridge Project. None were excluded due to their geographic location or ability to pay. Patients were excluded due severe clinical deterioration, weekend presentation with urgent need to start therapy, lack of sufficient specimen, or immediate transfer to a hospital with access to specialized diagnostic studies. The service outcomes help us evaluate the implementation outcomes of interest, including feasibility, utility, and appropriateness. See **Table 4** for details.

## 4 Discussion

By securing access to a standardized consensus-derived diagnostic panel, the Bridge Project has allowed better characterization of childhood ALL in Mexico while producing unprecedented service improvements and documenting key implementation outcomes. We have been able to prospectively apply the full diagnostic panel as part of routine care, support proper risk-group assignment, and document the frequency of classic molecular alterations, as well as the value of a carefully selected centralized approach. We are using these results to inform iterative changes to the diagnostic panel, support knowledge-transfer to additional reference laboratories, and solidify the multi-site expansion, implementation, and impact evaluation of an associated evidence-based consensus-derived ALL treatment guideline (MAS-ALL18).

Situational analyses conducted prior to launching the Bridge Project showed that while the access to diagnostic confirmation by immunophenotype was high (97%), access to specialized studies was limited; up to a third of patients with confirmed ALL lacked access to karyotype, DNA index, and MRD evaluation, and up to two-thirds of patients lacked access to FISH and PCR studies (18, 19). These and other findings identified during situational analyses, were recently confirmed and more thoroughly evaluated in an expanded cohort, including 2,116 patients <18 years of age diagnosed at sixteen Mexican institutions between 2011-2019 (34).

Through the Bridge Project, 100% of patients with suspected ALL at participating institutions had access to a timely and precise diagnostic evaluation, 98.2% patients with confirmed ALL benefited from application of the full day zero diagnostic panel, and an estimated 95.7% of patients with access to the day zero panel, gained access to it. Service delivery has improved in the following four quality dimensions: equity, timeliness, effectiveness, and patient-centeredness and the improvements have been achieved through a combination of consensus development, prioritization, centralization, standardization, optimization, and training. A multisite harmonized diagnostic approach has been achieved despite the Bridge Project not forcing institutions to apply a unified therapeutic protocol or approach (see **Supplemental Resource 4** for details). Additional details about the theory of change and measurement strategy, including change ideas, PDSA cycles, and time series charts (run and control) will be reported elsewhere. From an implementation standpoint, the feasibility, utility, and appropriateness of the diagnostic panel have also been established.

The Bridge Project has served as a demonstration project for the value of centralization and external validation of specialized studies – two concepts that were not being pursued when the project was conceived and are gaining acceptance in the pediatric hematology-oncology community in Mexico. The project is expanding to more referring institutions and two additional laboratories (a government-designated national flow cytometry laboratory and a government-designated national genomics laboratory). While organizing state-by-state or regional strategies is outside MAS’ scope and capability, conducting knowledge-transfer and incorporating these two national laboratories will extend the service and training opportunities to governmental laboratories and help expand training and external validation activities nationally. In addition, the training curriculum and external validation opportunities developed to conduct knowledge-transfer, will support local laboratories at MAS member institutions that procure equipment and want to engage in systematic local capacity and capability building activities in collaboration with other members of the cooperative group to offer these high-quality diagnostic panel services locally or regionally.

For risk-stratification, before the Bridge project, most patients (82%) were classified as high-risk, despite half of them not meeting NCI criteria for high-risk classification (19). Although are not able to report on preliminary or final risk stratification in this manuscript due to the multitude of modified treatment guidelines used by the institutions and the absence of WBC count in our service and billing datasets, Cohort 1 participating institutions utilizing MAS-ALL18 have looked at this in detail and noticed a shift towards a more classic risk-group distribution after introducing the diagnostic panel. For the first 137 patients utilizing MAS-ALL18, 49.5% of patients were classified as favorable, 20.8% as intermediate, and 29.7% as high-risk (21). Per discussion with participating institutions, the new risk-group distribution has been sustained as the project has continued. We are designing a hybrid type 3 effectiveness-implementation study that will retrospectively validate the diagnostic panel and prospectively assess its impact on proximal and distal clinical outcomes.

Through the Bridge Project, we also documented the frequency of classic molecular alterations in a larger sample of Mexican children with ALL than in past studies, which have historically included 53-298 Mexican nationals (35). Consistent with the literature, we observed a lower frequency of t(12;21)/ETV6-RUNX1 compared to non-Hispanics (15.0% in our cohort, compared to 8.4-14.9% in other Mexican and Hispanic cohorts and 24% in Non-Hispanic cohorts) in the United States (35). We also observed a higher frequency of iAMP21 (8.5%) in Mexicans, compared to Hispanics (1%) and Non-Hispanics (2%) (36, 37) and a higher frequency of t(1;19)/EA2-PBX1 (7.5%) compared to baseline studies in Non-Hispanics (5%) (38). EA2-PBX1 and iAMP21 findings are consistent with prior reports in Mexican patients, where EA2-PBX1 has been identified in 7.2% of patients and iAMP21 in up to 10% of patients (35). Our observed frequencies for t(4;11)/KMT2A and t(9;22)/BCR-ABL (4.2% and 3.2%, respectively) are consistent with the literature for all three ethnicities (36, 39). Finally, our observed frequency of hypodiploidy by IDNA (1.6%) is lower than reported in Mexican series, but consistent with the reported frequency in Hispanics and Non-Hispanics in the United States (1 and 2%), respectively (35, 36).

With regards to disease evaluation and downstream tests included in the diagnostic panel, during the Bridge Project, minimal residual disease (MRD) has been performed in 403 (87%) of 463 patients with B-cell ALL at day 15 and in 305 (65.9%) at day 84, and for 28 (67%) of 42 patients with T-cell ALL at day 29 and 22 (52.4%) at day 84. These frequencies are better than those identified during situational analyses, where MRD during induction was shown to be performed in only 61% of patients (19). However, they are lower than desirable. In HIC manuscripts <2% of new diagnosis patients lack MRD results (40). We first thought this was due to use of non-MRD based protocols because at the start of this project, the national protocol was based on Total XIIIB (a non-MRD protocol), but realized that explains a small fraction of cases. Based on additional situational analyses and recent abstracts, we hypothesized the lack of access to MRD evaluation likely resulted from a combination of early death (8-12%), treatment abandonment (2-6%), induction failure (1-2%), lack of clearance for the procedure due to toxicity or poor health status when MRD is due, and scheduling conflicts or misses (18, 19, 21). A sub-analysis on the distribution of causes for missed MRD confirmed death before MRD timepoints as the main contributor to missed MRD evaluation (affecting 3-24% of patients in this cohort, depending on the timepoint), followed by not utilizing the timepoint in the institutional guideline (specific to day 84), transfers to another facility, clinical instability, lack of coordination at site to ship the sample, and treatment abandonment (see **Supplemental Resource 3**). Although we did not aim to conduct a cancer outcomes analysis, understanding the reasons for missing MRD evaluation allowed us to document major challenges with mortality in the first 90 days for B-cell and T-cell patients, since 38/463 (8.2%) patients with B-cell ALL and 9/42 (21.4%) patients with T-cell ALL were reported as diseased before day 84 MRD evaluation (see **Supplemental Resource 3**). Finally, considering negative MRD a value <1% at day 15 and a value <0.01% at 84, 85.4% and 85.6% of B-cell ALL were documented to have negative MRD during induction and at the end of consolidation, which is consistent with the literature (40, 41).

The clinical, molecular, and response to therapy attributes observed in this cohort after securing access to a comprehensive diagnostic panel do not fully explain the rapid decline in survival (with ≥20% of patients not reaching day 15 in a clinical condition amenable to obtaining an MRD sample) and support what other authors have called the “triple-hit explanation” for worse ALL outcomes among Mexican and Hispanic children (35). The theory incorporates high incidence and burden of leukemia, higher frequency classic and novel adverse biologic features (such as iAMP21, t(1;19)/EA2-PBX1, CRLF2, TPMT, and NUDT15 mutations), and suboptimal treatment among Mexican and Hispanic children to explain the poor outcomes. To improve the understanding of factors influencing poor outcomes for children with ALL in Mexico, we introduced next-generation sequencing in the recently awarded 2022 Gonzalo Rio Arronte Foundation grant and have started collecting results. A comprehensive survival analysis is currently beyond the scope of the current version of the Bridge Project. However, as MAS-ALL18 expands, more detailed clinical annotation and a thorough survival analysis will be pursued. We also continue to support the large-scale implementation of a variety of evidence-based supportive care initiatives, as described in the MAS Strategy (**Figure 1**).

Two years designing and four years implementing the Bridge Project have offered many opportunities to discuss the role and value of centralization vs. decentralization in pediatric oncology-wise health systems. Decentralization offers the advantage of bringing care closer to where the patient and their family live, which can reduce treatment abandonment and the economic burden of disease. However, it also creates challenges by diluting resources and limiting access to volume-based expertise. Centralization offers the advantage of concentrated technology and expertise but poses challenges to the patients and families who live far away and have relevant competing priorities. However, in recent years, models for shared care have expanded and the tension between centralization and decentralization through the application of telemedicine and various levels of pediatric hematology/oncology care has increased (42, 43). In 2020, the Lancet Oncology Commission for Sustainable care for children with cancer proposed a facility levels and country tiers framework, as a mechanism to address and harmonize the approach to these challenges (5). In MAS, we have learned the value of multi-site collaboration and the implementation and optimization of a carefully selected centralized approach. As a result, we are expanding the project to incorporate two additional strategically located laboratories. In the case of diagnostic specimens that do not require the patient to travel, navigating the logistics so that every child with suspected ALL has the same opportunity of accessing a comprehensive diagnostic panel is worthwhile.

We conclude by addressing some of the limitations of this project. Given its emphasis on service improvement and implementation evaluation, we have not routinely collected all the clinical variables that would be of interest for a full ALL epidemiologic analysis and are not able to document the prognostic significance of the molecular findings. However, we are expanding the project as described above and expect to be able to generate this analysis in the future.

## Data availability statement

The datasets presented in this article are not readily available because in consideration of institutional privacy, requests for data will be considered on an individual basis. If agreed upon by all participating sites the deidentified datasets will be shared with the requestor. Datasets will be limited to data elements informing the publication. Requests to access the datasets should be directed to PF, paola.friedrich@stjude.org.

## Author contributions

PF: Conceptualization, Data curation, Formal analysis, Funding acquisition, Methodology, Supervision, Writing – original draft, Resources, Writing – review & editing. NM: Conceptualization, Funding acquisition, Methodology, Project administration, Writing – original draft, Writing – review & editing. NE-A: Conceptualization, Funding acquisition, Methodology, Project administration, Writing – original draft, Visualization, Resources, Writing – review & editing. KG-G: Conceptualization, Funding acquisition, Methodology, Project administration, Writing – review & editing, Visualization. MG-Z: Data curation, Investigation, Supervision, Writing – review & editing, Visualization. ML-R: Investigation, Supervision, Writing – review & editing. CP-Z: Investigation, Supervision, Writing – review & editing. LG-S: Investigation, Supervision, Writing – review & editing. MR-G: Investigation, Supervision, Writing – review & editing. NL-F: Investigation, Supervision, Writing – review & editing. DC-P: Investigation, Supervision, Writing – review & editing. MV-E: Investigation, Supervision, Writing – review & editing. AM-G: Investigation, Supervision, Writing – review & editing. DC-Z: Investigation, Supervision, Writing – review & editing. LV-C: Investigation, Supervision, Writing – review & editing. JL-O: Investigation, Supervision, Writing – review & editing. SG-S: Data curation, Investigation, Writing – review & editing. JM-S: Data curation, Investigation, Writing – review & editing. PM-S: Data curation, Investigation, Writing – review & editing. PC-T: Data curation, Investigation, Writing – review & editing. AS-C: Data curation, Investigation, Writing – review & editing. IG-M: Data curation, Investigation, Writing – review & editing. MJ-O: Data curation, Investigation, Writing – review & editing. DA-C: Conceptualization, Data curation, Investigation, Supervision, Validation, Writing – review & editing. DA-E: Conceptualization, Data curation, Investigation, Supervision, Validation, Writing – review & editing. PG-M: Conceptualization, Data curation, Investigation, Supervision, Validation, Writing – review & editing. HR-R: Conceptualization, Data curation, Investigation, Supervision, Validation, Writing – review & editing.

## Supplementary material

Supplemental Resource 1 Diagnostic Panel Costs *MRD: Minimal Residual Disease. For B-cell ALL MRD is measured as MRD Lite on Day 15 and only repeated on Day 29 if there is concern for induction failure. For T-cell ALL, MRD is measured as Full MRD on Day 29. Cost is shown with only two MRD evaluations because it typically occurs at two time points (Day 15 or Day 29, and Day 84). **Costs are reported in Mexican Pesos (MXN) and converted to US Dollars (USD) using the average currency conversion from July 2018 to July 2023 reported by the Bank of Mexico, 1 USD = 20.0 MXN Peso.

Supplemental Resource 2 Sample Arrival Checklist (version 04.11.19).

Supplemental Resource 3 Evaluation of Missing MRD results *Totals throughout table are n=463 for B-cell ALL and n=42 for T-cell ALL.

Supplemental Resource 4 Pre-approved bone marrow samples vs. to request by age, lineage and protocol.
