# Supplementary material for: Securing access to a comprehensive diagnostic panel for children with suspected acute lymphoblastic leukemia: Results from the Mexico in Alliance with St. Jude “Bridge Project”
Source: Front Oncol. 2024 Jan 15;13:1286278. doi: 10.3389/fonc.2023.1286278 (PMC10824571; doi:10.3389/fonc.2023.1286278)
Supplement: Supplemental Resource 1 — Diagnostic Panel Costs *MRD: Minimal Residual Disease. For B-cell ALL MRD is measured as MRD Lite on Day 15 and only repeated on Day 29 if there is concern for induction failure. For T-cell ALL, MRD is measured as Full MRD on Day 29. Cost is shown with only two MRD evaluations because it typically occurs at two time points (Day 15 or Day 29, and Day 84). **Costs are reported in Mexican Pesos (MXN) and converted to US Dollars (USD) using the average currency conversion from July 2018 to July 2023 reported by the Bank of Mexico, 1 USD = 20.0 MXN Peso. [file DataSheet_1.pdf]

Supplemental Materials

Securing access to a comprehensive diagnostic panel for children with suspected acute lymphoblastic leukemia: Results from the Mexico in Alliance with St. Jude "Bridge Project"

Friedrich P, et al. Frontiers 2023

Supplemental Resource 1. Diagnostic Panel Costs

Phase	Diagnostic Panel	Costs July 2019 - September 2022**	Costs as of October 2022**
Diagnosis	Immunophenotype	$10,362 MXN	$7,760 MXN
Diagnosis	FISH	$8,328 MXN	$8,328 MXN
Diagnosis	Karyotype	$7,767 MXN	$6,379 MXN
Diagnosis	DNA Index	$2,220 MXN	$1,705 MXN
Monitoring	MRD, Day 15 (or Day 29*)	$5,181 MXN	$4,741 MXN
Monitoring	MRD, Day 84	$5,181 MXN	$4,741 MXN
	Total in MXN	$39,040 MXN	$33,654.00 MXN
	Total in USD	$ 1,952 USD	$ 1,682.7 USD

*MRD: Minimal Residual Disease. For B-cell ALL MRD is measured as MRD Lite on Day 15 and only repeated on Day 29 if there is concern for induction failure. For T-cell ALL, MRD is measured as Full MRD on Day 29. Cost is shown with only two MRD evaluations because it typically occurs at two time points (Day 15 or Day 29, and Day 84).
**Costs are reported in Mexican Pesos (MXN) and converted to US Dollars (USD) using the average currency conversion from July 2018 to July 2023 reported by the Bank of Mexico, 1 USD = 20.0 MXN Peso.

Supplemental Resource 2. Sample Arrival Checklist (version 04.11.19)

Patient Code: ____
Sample Date: ______________________________
Sample Time (LOCAL TIME): _________________________
MILESTONE Sample Receipt Date: ________________________
MILESTONE Sample Receipt Time: ________________________

**Package Reception**	**Yes= 1 / No= 0**	**Provide Feedback**
**Did it arrive within 48 hours? (Consider time zone differences)**	Critical	<48 accepted<br>>48 rejected
**Was email sent with complete data?**		
**Was a full clinical summary sent in the package?**		
**Was lab application submitted complete in the package and correctly filled out?**		
**Did the secondary container arrive intact?**		
**Is the temperature inside the secondary container adequate?**	Critical	

**Primary Vessel Evaluation**		**Purple 1 (Yes = 1 / No = 0)**	**Purple 2 (Yes= 1/No= 0**	**Green 1 (Yes = 1 / No = 0)**	**Green 2 (Yes = 1 / No = 0)**
**Correct Primary Container (Green/Purple Tubes)**					
**Correct labeling**					
**Adequate sample volume**	1-2 ml conditioned<br><1 ml rejected				
**Sample without clots**	decide based on the size of the clot				
**Sample without hemolysis**					
**Sample without spillage**	decide based on the amount of spillage				
**Status (accepted, conditional, rejected)**					

**Evaluation of slides**	**(Yes = 1 / No = 0) NA: No applies**	**Feedback**
**Contains 2 dyed slides**		
**Smear suitable for reading**		
**Smear stain suitable for reading**		
**Contains 4 undyed sipes**		
**Touch prep suitable (SI/NO or N/A)**		

**Results Submission Commitment Date**	
**Smear**	
**IDNA**	
**Karyotype**	
**Immunophenotype**	
**FISH**	
**MRD**	

**Validated:**			
**Area:**	Cytometry/Molecular Biology	Cytogenetics	Morphology

Supplemental Resource 3: Evaluation of Missing MRD results

	MRD D15		MRD D29		MRD D84	
	YES	NO	YES	NO	YES	NO
**B cell ALL**<br>(n= 463 patients)	**403**<br>**87%**	**60**<br>**13%**	**9**<br>**1.9%**		**305**<br>**65.9%**	**158**<br>**34.1%**
**T cell ALL**<br>(n= 42 patients)	**4**<br>**9.5%**		**28**<br>**66.7%**	**14**<br>**33.3%**	**22**<br>**52.4%**	**20**<br>**47.6%**

*412 (88.9%) of 463 patients with B cell ALL had MRD performed at Day 15 or 29 and 305 (65.8%) at Day 84.

**32 (76.1%) of 42 patients with T cell ALL had MRD performed at Day 15 or 29 or 15 and 22 (52.4%) at day 84.

	**Missing MRD 15 B-cell**		**Missing MRD 84 B-cell**		**Missing MRD 29 T-cell**		**Missing MRD 84 T-cell**	
**REASONS / % of TOTALS***	**60**	**13%**	**158**	**34.1%**	**14**	**33.3%**	**20**	**47.6%**
**Patient died before MRD**	**14**	**3.0%**	**38**	**8.2%**	**7**	**16.7%**	**9**	**21.4%**
**Transferred to another unit of care**	**12**	**2.6%**	**17**	**3.7%**	**1**	**2.4%**	**2**	**4.8%**
**Clinical Instability**	**11**	**2.4%**	10	2.2%	0	0.0%	1	2.4%
Institutional guideline does not recognize the MRD timepoint	0	0.0%	**42**	**9.1%**	0	0.0%	**3**	**7.1%**
Lack of coordination at site to ship sample	6	1.3%	**11**	**2.4%**	**2**	**4.8%**	**3**	**7.1%**
Treatment Abandonment	5	1.1%	**11**	**2.4%**	0	0.0%	**1**	**2.4%**
Does not meet time to send sample	0	0.0%	8	1.7%	0	0.0%	0	0.0%
Other / Unknown reason	12	2.6%	21	4.5%	4	9.5%	1	2.4%

*Totals throughout table are n=463 for B-cell ALL and n=42 for T-cell ALL.

Supplemental Resource 4. Pre-approved bone marrow samples vs. to request by age, lineage and protocol

	Pre-approved by Río Arronte grant												Require approval by CDLA or other				
	Morphology BMA	Immunophenotype	Karyotype	IDNA	FISH BCR-ABL	FISH ETV6-RUNX1	FISH MLL-AF4	FISH EA2-PBX1	FISH iAMP21	MRD Day 15 (Lite)	MRD Day 29* (Full) – IND	MRD Day 84* (Full) – CONS	MRD MLL (PCR-Q) – IND	MRD MLL (PCR-Q) – CONS	MRD Ph^+ (PCR-Q) – IND	MRD Ph^+ (PCR-Q) – CONS	MRD Ph^+ (PCR-Q) – 6 mo.
Age 0-1 (<365 days)	X	X	X	X	X	X	X	X	X	X	X	X	X	X			
Age 1-18, Total XV modified																	
Strain B	X	X	X	X	X	X	X	X	X	X	X	X					
Strain T	X	X	X	X	X		X				X	X					
Age 1-18, XIIIA/B (Old National Protocol)																	
Strain B	X	X	X	X	X	X	X	X	X								
Strain T	X	X	X	X	X		X										
Age 1-18, Tijuana																	
Strain B (modified COG-BFM)	X	X	X	X	X	X	X	X	X	X	X						
Strain T (modified COG)	X	X	X	X	X	X	X	X	X		X	X^{Δ}					
Age 1-18, MAS-ALL18 Scheme (in patient not eligible for protocol)																	
Strain B	X	X	X	X	X	X	X	X	X	X	X	X					
Strain T	X	X	X	X	X		X				X	X					
Age 1-18, any protocol																	
Ph + (BCR-ABL) by FISH															X	X	X
MLL^+ by FISH													X	X			

Lite = "Lite" technique flow cytometry; Full = Flow cytometry with complete technique. IND = End of induction; CONS = End of consolidation; PCR = Polymerase chain reaction - Quantitative. CDLA = House of Friendship. Prot. Nac. = National Protocol.
* Day 29 or equivalent to "end of induction"; Day 84 or equivalent to "end of consolidation" depending on protocol terminology.
Δ Only if the result was positive on day 29.
